# Critical Thinking, Intelligence, and Unsubstantiated Beliefs: An Integrative Review

**DOI:** 10.3390/jintelligence11110207

**Published:** 2023-10-30

**Authors:** D. Alan Bensley

**Affiliations:** Department of Psychology, Frostburg State University, Frostburg, MD 21532, USA; abensley@frostburg.edu

**Keywords:** critical thinking, intelligence, cognitive ability, dispositions, unsubstantiated beliefs

## Abstract

A review of the research shows that critical thinking is a more inclusive construct than intelligence, going beyond what general cognitive ability can account for. For instance, critical thinking can more completely account for many everyday outcomes, such as how thinkers reject false conspiracy theories, paranormal and pseudoscientific claims, psychological misconceptions, and other unsubstantiated claims. Deficiencies in the components of critical thinking (in specific reasoning skills, dispositions, and relevant knowledge) contribute to unsubstantiated belief endorsement in ways that go beyond what standardized intelligence tests test. Specifically, people who endorse unsubstantiated claims less tend to show better critical thinking skills, possess more relevant knowledge, and are more disposed to think critically. They tend to be more scientifically skeptical and possess a more rational–analytic cognitive style, while those who accept unsubstantiated claims more tend to be more cynical and adopt a more intuitive–experiential cognitive style. These findings suggest that for a fuller understanding of unsubstantiated beliefs, researchers and instructors should also assess specific reasoning skills, relevant knowledge, and dispositions which go beyond what intelligence tests test.

## 1. Introduction

Why do some people believe implausible claims, such as the QAnon conspiracy theory, that a cabal of liberals is kidnapping and trafficking many thousands of children each year, despite the lack of any credible supporting evidence? Are believers less intelligent than non-believers? Do they lack knowledge of such matters? Are they more gullible or less skeptical than non-believers? Or, more generally, are they failing to think critically?

Understanding the factors contributing to acceptance of unsubstantiated claims is important, not only to the development of theories of intelligence and critical thinking but also because many unsubstantiated beliefs are false, and some are even dangerous. Endorsing them can have a negative impact on an individual and society at large. For example, false beliefs about the COVID-19 pandemic, such as believing that 5G cell towers induced the spread of the COVID-19 virus, led some British citizens to set fire to 5G towers (Jolley and Paterson 2020). Other believers in COVID-19 conspiracy theories endangered their own and their children’s lives when they refused to socially distance and be vaccinated with highly effective vaccines, despite the admonitions of scientific experts (Bierwiaczonek et al. 2020). Further endangering the population at large, those who believe the false conspiracy theory that human-caused global warming is a hoax likely fail to respond adaptively to this serious global threat (van der Linden 2015). Parents, who uncritically accept pseudoscientific claims, such as the false belief that facilitated communication is an effective treatment for childhood autism, may forego more effective treatments (Lilienfeld 2007). Moreover, people in various parts of the world still persecute other people whom they believe are witches possessing supernatural powers. Likewise, many people still believe in demonic possession, which has been associated with mental disorders (Nie and Olson 2016). Compounding the problems created by these various unsubstantiated beliefs, numerous studies now show that when someone accepts one of these types of unfounded claims, they tend to accept others as well; see Bensley et al. (2022) for a review.

Studying the factors that contribute to unfounded beliefs is important not only because of their real-world consequences but also because this can facilitate a better understanding of unfounded beliefs and how they are related to critical thinking and intelligence. This article focuses on important ways in which critical thinking and intelligence differ, especially in terms of how a comprehensive model of CT differs from the view of intelligence as general cognitive ability. I argue that this model of CT more fully accounts for how people can accurately decide if a claim is unsubstantiated than can views of intelligence, emphasizing general cognitive ability. In addition to general cognitive ability, thinking critically about unsubstantiated claims involves deployment of specific reasoning skills, dispositions related to CT, and specific knowledge, which go beyond the contribution of general cognitive ability.

Accordingly, this article begins with an examination of the constructs of critical thinking and intelligence. Then, it discusses theories proposing that to understand thinking in the real world requires going beyond general cognitive ability. Specifically, the focus is on factors related to critical thinking, such as specific reasoning skills, dispositions, metacognition, and relevant knowledge. I review research showing that that this alternative multidimensional view of CT can better account for individual differences in the tendency to endorse multiple types of unsubstantiated claims than can general cognitive ability alone.

## 2. Defining Critical Thinking and Intelligence

Critical thinking is an almost universally valued educational objective in the US and in many other countries which seek to improve it. In contrast, intelligence, although much valued, has often been viewed as a more stable characteristic and less amenable to improvement through specific short-term interventions, such as traditional instruction or more recently through practice on computer-implemented training programs. According to Wechsler’s influential definition, intelligence is a person’s “aggregate or global capacity to act purposefully, to think rationally, and to deal effectively with his environment” (Wechsler 1944, p. 3).

Consistent with this definition, intelligence has long been associated with general cognitive or intellectual ability and the potential to learn and reason well. Intelligence (IQ) tests measure general cognitive abilities, such as knowledge of words, memory skills, analogical reasoning, speed of processing, and the ability to solve verbal and spatial problems. General intelligence or “g” is a composite of these abilities statistically derived from various cognitive subtests on IQ tests which are positively intercorrelated. There is considerable overlap between g and the concept of fluid intelligence (Gf) in the prominent Cattell–Horn–Carroll model (McGrew 2009), which refers to “the ability to solve novel problems, the solution of which does not depend on previously acquired skills and knowledge,” and crystalized intelligence (Gc), which refers to experience, existing skills, and general knowledge (Conway and Kovacs 2018, pp. 50–51). Although g or general intelligence is based on a higher order factor, inclusive of fluid and crystallized intelligence, it is technically not the same as general cognitive ability, a commonly used, related term. However, in this article, I use “general cognitive ability” and “cognitive ability” because they are the imprecise terms frequently used in the research reviewed.

Although IQ scores have been found to predict performance in basic real-world domains, such as academic performance and job success (Gottfredson 2004), an enduring question for intelligence researchers has been whether g and intelligence tests predict the ability to adapt well in other real-world situations, which concerns the second part of Wechsler’s definition. So, in addition to the search for the underlying structure of intelligence, researchers have been perennially concerned with how general abilities associated with intelligence can be applied to help a person adapt to real-world situations. The issue is largely a question of how cognitive ability and intelligence can help people solve real-world problems and cope adaptively and succeed in dealing with various environmental demands (Sternberg 2019).

Based on broad conceptual definitions of intelligence and critical thinking, both intelligence and CT should aid adaptive functioning in the real world, presumably because they both involve rational approaches. Their common association with rationality gives each term a positive connotation. However, complicating the definition of each of these is the fact that rationality also continues to have a variety of meanings. In this article, in agreement with Stanovich et al. (2018), rationality is defined in the normative sense, used in cognitive science, as the distance between a person’s response and some normative standard of optimal behavior. As such, degree of rationality falls on a continuous scale, not a categorical one.

Despite disagreements surrounding the conceptual definitions of intelligence, critical thinking, and rationality, a commonality in these terms is they are value-laden and normative. In the case of intelligence, people are judged based on norms from standardized intelligence tests, especially in academic settings. Although scores on CT tests seldom are, nor could be, used to judge individuals in this way, the normative and value-laden basis of CT is apparent in people’s informal judgements. They often judge others who have made poor decisions to be irrational or to have failed to think critically.

This value-laden aspect of CT is also apparent in formal definitions of CT. Halpern and Dunn (2021) defined critical thinking as “the use of those cognitive skills or strategies that increase the probability of a desirable outcome. It is used to describe thinking that is purposeful, reasoned, and goal-directed.” The positive conception of CT as helping a person adapt well to one’s environment is clearly implied in “desirable outcome”.

Robert Ennis (1987) has offered a simpler, yet useful definition of critical thinking that also has normative implications. According to Ennis, “critical thinking is reasonable, reflective thinking focused on deciding what to believe or do” (Ennis 1987, p. 102). This definition implies that CT helps people know what to believe (a goal of epistemic rationality) and how to act (a goal of instrumental rationality). This is conveyed by associating “critical thinking” with the positive terms, “reasonable” and “reflective”. Dictionaries commonly define “reasonable” as “rational”, “logical”, “intelligent”, and “good”, all terms with positive connotations.

For critical thinkers, being reasonable involves using logical rules, standards of evidence, and other criteria that must be met for a product of thinking to be considered good. Critical thinkers use these to evaluate how strongly reasons or evidence supports one claim versus another, drawing conclusions which are supported by the highest quality evidence (Bensley 2018). If no high-quality evidence is available for consideration, it would be unreasonable to draw a strong conclusion. Unfortunately, people’s beliefs are too often based on acceptance of unsubstantiated claims. This is a failure of CT, but is it also a failure of intelligence?

## 3. Does Critical Thinking “Go Beyond” What Is Meant by Intelligence?

Despite the conceptual overlap in intelligence and CT at a general level, one way that CT can be distinguished from the common view of intelligence as general cognitive ability is in terms of what each can account for. Although intelligence tests, especially measures of general cognitive ability, have reliably predicted academic and job performance, they may not be sufficient to predict other everyday outcomes for which CT measures have made successful predictions and have added to the variance accounted for in performance. For instance, replicating a study by Butler (2012), Butler et al. (2017) obtained a negative correlation (*r* = −0.33) between scores on the Halpern Critical Thinking Appraisal (HCTA) and a measure of 134 negative, real-world outcomes, not expected to befall critical thinkers, such as engaging in unprotected sex or posting a message on social media which the person regretted. They found that higher HCTA scores not only predicted better life decisions, but also predicted better performance beyond a measure of general cognitive ability. These results suggest that CT can account for real-world outcomes and goes beyond general cognitive ability to account for additional variance.

Some theorists maintain that standardized intelligence tests do not capture the variety of abilities that people need to adapt well in the real world. For example, Gardner (1999), has proposed that additional forms of intelligence are needed, such as spatial, musical, and interpersonal intelligences in addition to linguistic and logical–mathematical intelligences, more typically associated with general cognitive ability and academic success. In other theorizing, Sternberg (1988) has proposed three additional types of intelligence: analytical, practical, and creative intelligence, to more fully capture the variety of intelligent abilities on which people differ. Critical thinking is considered part of analytical skills which involve evaluating the quality and applicability of ideas, products, and options (Sternberg 2022). Regarding adaptive intelligence, Sternberg (2019) has emphasized how adaptive aspects of intelligence are needed to solve real-world problems both at the individual and species levels. According to Sternberg, core components of intelligence have evolved in humans, but intelligence takes different forms in different cultures, with each culture valuing its own skills for adaptation. Thus, the construct of intelligence must go beyond core cognitive ability to encompass the specific abilities needed for adaptive behavior in specific cultures and settings.

Two other theories propose that other components be added to intelligent and rational thinking. Ackerman (2022) has emphasized the importance of acquiring domain-specific knowledge for engaging in intelligent functioning in the wide variety of tasks found in everyday life. Ackerman has argued that declarative, procedural, and tacit knowledge, as well as non-ability variables, are needed to better predict job performance and performance of other everyday activities. Taking another approach, Halpern and Dunn (2021) have proposed that critical thinking is essentially the adaptive application of intelligence for solving real-world problems. Elsewhere, Butler and Halpern (2019) have argued that dispositions such as open-mindedness are another aspect of CT and that domain-specific knowledge and specific CT skills are needed to solve real-world problems.

Examples are readily available for how CT goes beyond what IQ tests test to include specific rules for reasoning and relevant knowledge needed to execute real-world tasks. Take the example of scientific reasoning, which can be viewed as a specialized form of CT. Drawing a well-reasoned inductive conclusion about a theory or analyzing the quality of a research study both require that a thinker possess relevant specialized knowledge related to the question and specific reasoning skills for reasoning about scientific methodology. In contrast, IQ tests are deliberately designed to be nonspecialized in assessing Gc, broadly sampling vocabulary and general knowledge in order to be fair and unbiased (Stanovich 2009). Specialized knowledge and reasoning skills are also needed in non-academic domains. Jurors must possess specialized knowledge to understand expert, forensic testimony and specific reasoning skills to interpret the law and make well-reasoned judgments about a defendant’s guilt or innocence.

Besides lacking specific reasoning skills and domain-relevant knowledge, people may fail to think critically because they are not disposed to use their reasoning skills to examine such claims and want to preserve their favored beliefs. Critical thinking dispositions are attitudes or traits that make it more likely that a person will think critically. Theorists have proposed numerous CT dispositions (e.g., Bensley 2018; Butler and Halpern 2019; Dwyer 2017; Ennis 1987). Some commonly identified CT dispositions especially relevant to this discussion are open-mindedness, skepticism, intellectual engagement, and the tendency to take a reflective, rational–analytic approach. Critical thinking dispositions are clearly value-laden and prescriptive. A good thinker should be open-minded, skeptical, reflective, intellectually engaged, and value a rational–analytic approach to inquiry. Conversely, corresponding negative dispositions, such as “close-mindedness” and “gullibility”, could obstruct CT.

Without the appropriate disposition, individuals will not use their reasoning skills to think critically about questions. For example, the brilliant mystery writer, Sir Arthur Conan Doyle, who was trained as a physician and created the hyper-reasonable detective Sherlock Holmes, was not disposed to think critically about some unsubstantiated claims. Conan Doyle was no doubt highly intelligent in cognitive ability terms, but he was not sufficiently skeptical (disposed to think critically) about spiritualism. He believed that he was talking to his dearly departed son though a medium, despite the warnings of his magician friend, Harry Houdini, who told him that mediums used trickery in their seances. Perhaps influenced by his Irish father’s belief in the “wee folk”, Conan Doyle also believed that fairies inhabited the English countryside, based on children’s photos, despite the advice of experts who said the photos could be faked. Nevertheless, he was skeptical of a new theory of tuberculosis proposed by Koch when he reported on it, despite his wife suffering from the disease. So, in professional capacities, Conan Doyle used his CT skills, but in certain other domains for which he was motivated to accept unsubstantiated claims, he failed to think critically, insufficiently disposed to skeptically challenge certain implausible claims.

This example makes two important points. Conan Doyle’s superior intelligence was not enough for him to reject implausible claims about the world. In general, motivated reasoning can lead people, even those considered highly intelligent, to accept claims with no good evidentiary support. The second important point is that we would not be able to adequately explain cases like this one, considering only the person’s intelligence or even their reasoning skills, without also considering the person’s disposition. General cognitive ability alone is not sufficient, and CT dispositions should also be considered.

Supporting this conclusion, Stanovich and West (1997) examined the influence of dispositions beyond the contribution of cognitive ability on a CT task. They gave college students an argument evaluation test in which participants first rated their agreement with several claims about real social and political issues made by a fictitious person. Then, they gave them evidence against each claim and finally asked them to rate the quality of a counterargument made by the same fictitious person. Participants’ ratings of the counterarguments were compared to the median ratings of expert judges on the quality of the rebuttals. Stanovich and West also administered a new measure of rational disposition called the Actively Open-minded Thinking (AOT) scale and the SAT as a proxy for cognitive ability. The AOT was a composite of items from several other scales that would be expected to measure CT disposition. They found that both SAT and AOT scores were significant predictors of higher argument analysis scores. Even after partialing out cognitive ability, actively open-minded thinking was significant. These results suggest that general cognitive ability alone was not sufficient to account for thinking critically about real-world issues and that CT disposition was needed to go beyond it.

Further examining the roles of CT dispositions and cognitive ability on reasoning, Stanovich and West (2008) studied myside bias, a bias in reasoning closely related to one-sided thinking and confirmation bias. A critical thinker would be expected to not show myside bias and instead fairly evaluate evidence on all sides of a question. Stanovich and West (2007) found that college students often showed myside bias when asked their opinions about real-world policy issues, such as those concerning the health risks of smoking and drinking alcohol. For example, compared to non-smokers, smokers judged the health risks of smoking to be lower. When they divided participants into higher versus lower cognitive ability groups based on SAT scores, the two groups showed little difference on myside bias. Moreover, on the hazards of drinking issue, participants who drank less had higher scores on the CT disposition measure.

Other research supports the need for both reasoning ability and CT disposition in predicting outcomes in the real world. Ren et al. (2020) found that CT disposition, as measured by a Chinese critical thinking disposition inventory, and a CT skill measure together contributed a significant amount of the variance in predicting academic performance beyond the contribution of cognitive ability alone, as measured by a test of fluid intelligence. Further supporting the claim that CT requires both cognitive ability and CT disposition, Ku and Ho (2010) found that a CT disposition measure significantly predicted scores on a CT test beyond the significant contribution of verbal intelligence in high school and college students from Hong Kong.

The contribution of dispositions to thinking is related to another way that CT goes beyond the application of general cognitive ability, i.e., by way of the motivation for reasoning. Assuming that all reasoning is motivated (Kunda 1990), then CT is motivated, too, which is implicit within the Halpern and Dunn (2021) and Ennis (1987) definitions. Critical thinking is motivated in the sense of being purposeful and directed towards the goal of arriving at an accurate conclusion. For instance, corresponding to pursuit of the goal of accurate reasoning, the CT disposition of “truth-seeking” guides a person towards reaching the CT goal of arriving at an accurate conclusion.

Also, according to Kunda (1990), a second type of motivated reasoning can lead to faulty conclusions, often by directing a person towards the goal of maintaining favored beliefs and preconceptions, as in illusory correlation, belief perseverance, and confirmation bias. Corresponding to this second type, negative dispositions, such as close-mindedness and self-serving motives, can incline thinkers towards faulty conclusions. This is especially relevant in the present discussion because poorer reasoning, thinking errors, and the inappropriate use of heuristics are related to the endorsement of unsubstantiated claims, all of which are CT failures. The term “thinking errors” is a generic term referring to logical fallacies, informal reasoning fallacies, argumentation errors, and inappropriate uses of cognitive heuristics (Bensley 2018). Heuristics are cognitive shortcuts, commonly used to simplify judgment tasks and reduce mental effort. Yet, when used inappropriately, heuristics often result in biased judgments.

Stanovich (2009) has argued that IQ tests do not test people’s use of heuristics, but heuristics have been found to be negatively correlated with CT performance (West et al. 2008). In this same study, they found that college students’ cognitive ability, as measured by performance on the SAT, was not correlated with thinking biases associated with use of heuristics. Although Stanovich and West (2008) found that susceptibility to biases, such as the conjunction fallacy, framing effect, base-rate neglect, affect bias, and myside bias were all uncorrelated with cognitive ability (using SAT as a proxy), other types of thinking errors were correlated with SAT.

Likewise, two types of knowledge are related to the two forms of motivated reasoning. For instance, inaccurate knowledge, such as misconceptions, can derail reasoning from moving towards a correct conclusion, as in when a person reasons from false premises. In contrast, reasoning from accurate knowledge is more likely to produce an accurate conclusion. Taking into account inaccurate knowledge and thinking errors is important to understanding the endorsement of unsubstantiated claims because these are also related to negative dispositions, such as close-mindedness and cynicism, none of which are measured by intelligence tests.

Critical thinking questions are often situated in real-world examples or in simulations of them which are designed to detect thinking errors and bias. As described in Halpern and Butler (2018), an item like one on the “Halpern Critical Thinking Assessment” (HCTA) provides respondents with a mock newspaper story about research showing that first-graders who attended preschool were better able to learn how to read. Then the question asks if preschool should be made mandatory. A correct response to this item requires recognizing that correlation does not imply causation, that is, avoiding a common reasoning error people make in thinking about research implications in everyday life. Another CT skills test, “Analyzing Psychological Statements” (APS) assesses the ability to recognize thinking errors and apply argumentation skills and psychology to evaluate psychology-related examples and simulations of real-life situations (Bensley 2021). For instance, besides identifying thinking errors in brief samples of thinking, questions ask respondents to distinguish arguments from non-arguments, find assumptions in arguments, evaluate kinds of evidence, and draw a conclusion from a brief psychological argument. An important implication of the studies just reviewed is that efforts to understand CT can be further informed by assessing thinking errors and biases, which, as the next discussion shows, are related to individual differences in thinking dispositions and cognitive style.

## 4. Dual-Process Theory Measures and Unsubstantiated Beliefs

Dual-process theory (DPT) and measures associated with it have been widely used in the study of the endorsement of unsubstantiated beliefs, especially as they relate to cognitive style. According to a cognitive style version of DPT, people have two modes of processing, a fast intuitive–experiential (I-E) style of processing and a slower, reflective, rational–analytic (R-A) style of processing. The intuitive cognitive style is associated with reliance on hunches, feelings, personal experience, and cognitive heuristics which simplify processing, while the R-A cognitive style is a reflective, rational–analytic style associated with more elaborate and effortful processing (Bensley et al. 2022; Epstein 2008). As such, the rational–analytic cognitive style is consistent with CT dispositions, such as those promoting the effortful analysis of evidence, objective truth, and logical consistency. In fact, CT is sometimes referred to as “critical-analytic” thinking (Byrnes and Dunbar 2014) and has been associated with analytical intelligence Sternberg (1988) and with rational thinking, as discussed before.

People use both modes of processing, but they show individual differences in which mode they tend to rely upon, although the intuitive–experiential mode is the default (Bensley et al. 2022; Morgan 2016; Pacini and Epstein 1999), and they accept unsubstantiated claims differentially based on their predominate cognitive style (Bensley et al. 2022; Epstein 2008). Specifically, individuals who rely more on an I-E cognitive style tend to endorse unsubstantiated claims more strongly, while individuals who rely more on a R-A cognitive style tend to endorse those claims less. Note, however, that other theorists view the two processes and cognitive styles somewhat differently, (e.g., Kahneman 2011; Stanovich et al. 2018).

Researchers have often assessed the contribution of these two cognitive styles to endorsement of unsubstantiated claims, using variants of three measures: the Cognitive Reflection Test (CRT) of Frederick (2005), the Rational–Experiential Inventory of Epstein and his colleagues (Pacini and Epstein 1999), and the related Need for Cognition scale of Cacioppo and Petty (1982). The CRT is a performance-based test which asks participants to solve problems that appear to require simple mathematical calculations, but which actually require more reflection. People typically do poorly on the CRT, which is thought to indicate reliance on an intuitive cognitive style, while better performance is thought to indicate reliance on the slower, more deliberate, and reflective cognitive style. The positive correlation of the CRT with numeracy scores suggests it also has a cognitive skill component (Patel et al. 2019). The Rational–Experiential Inventory (REI) of Pacini and Epstein (1999) contains one scale designed to measure an intuitive–experiential cognitive style and a second scale intended to measure a rational–analytic (R-A) style. The R-A scale was adapted from the Need for Cognition (NFC) scale of Cacioppo and Petty (1982), another scale associated with rational–analytic thinking and expected to be negatively correlated with unsubstantiated beliefs. The NFC was found to be related to open-mindedness and intellectual engagement, two CT dispositions (Cacioppo et al. 1996).

The cognitive styles associated with DPT also relate to CT dispositions. Thinking critically requires that individuals be disposed to use their reasoning skills to reject unsubstantiated claims (Bensley 2018) and that they be inclined to take a rational–analytic approach rather than relying on their intuitions and feelings. For instance, Bensley et al. (2014) found that students who endorsed more psychological misconceptions adopted a more intuitive cognitive style, were less disposed to take a rational–scientific approach to psychology, and scored lower on a psychological critical thinking skills test. Further supporting this connection, West et al. (2008) found that participants who tended to use cognitive heuristics more, thought to be related to intuitive processing and bias, scored lower on a critical thinking measure. As the Bensley et al. (2014) results suggest, in addition to assessing reasoning skills and dispositions, comprehensive CT assessment research should assess knowledge and unsubstantiated beliefs because these are related to failures of critical thinking.

## 5. Assessing Critical Thinking and Unsubstantiated Beliefs

Assessing endorsement of unsubstantiated claims provides another way to assess CT outcomes related to everyday thinking, which goes beyond what intelligence tests test (Bensley and Lilienfeld 2020). From the perspective of the multi-dimensional model of CT, endorsement of unsubstantiated claims could result from deficiencies in a person’s CT reasoning skills, a lack of relevant knowledge, and in the engagement of inappropriate dispositions. Suppose an individual endorses an unsubstantiated claim, such as believing the conspiracy theory that human-caused global warming is a hoax. The person may lack the specific reasoning skills needed to critically evaluate the conspiracy. Lantian et al. (2020) found that scores on a CT skills test were negatively correlated with conspiracy theory beliefs. The person also must possess relevant scientific knowledge, such as knowing the facts that each year humans pump about 40 billion metric tons of carbon dioxide into the atmosphere and that carbon dioxide is a greenhouse gas which traps heat in the atmosphere. Or, the person may not be scientifically skeptical or too cynical or mistrustful of scientists or governmental officials.

Although endorsing unsubstantiated beliefs is clearly a failure of CT, problems arise in deciding which ones are unsubstantiated, especially when considering conspiracy theories. Typically, the claims which critical thinkers should reject as unsubstantiated are those which are not supported by objective evidence. But of the many conspiracies proposed, few are vigorously examined. Moreover, some conspiracy theories which authorities might initially deny turn out to be real, such as the MK-Ultra theory that the CIA was secretly conducting mind-control research on American citizens.

A way out of this quagmire is to define unsubstantiated beliefs on a continuum which depends on the quality of evidence. This has led to the definition of unsubstantiated claims as assertions which have not been supported by high-quality evidence (Bensley 2023). Those which are supported have the kind of evidentiary support that critical thinkers are expected to value in drawing reasonable conclusions. Instead of insisting that a claim must be demonstrably false to be rejected, we adopt a more tentative acceptance or rejection of claims, based on how much good evidence supports them. Many claims are unsubstantiated because they have not yet been carefully examined and so totally lack support or they may be supported only by low quality evidence such as personal experience, anecdotes, or non-scientific authority. Other claims are more clearly unsubstantiated because they contradict the findings of high-quality research. A critical thinker should be highly skeptical of these.

Psychological misconceptions are one type of claim that can be more clearly unsubstantiated. Psychological misconceptions are commonsense psychological claims (folk theories) about the mind, brain, and behavior that are contradicted by the bulk of high-quality scientific research. Author developed the Test of Psychological Knowledge and Misconceptions (TOPKAM), a 40-item, forced-choice measure with each item posing a statement of a psychological misconception and the other response option stating the evidence-based alternative (Bensley et al. 2014). They found that higher scores on the APS, the argument analysis test applying psychological concepts to analyze real-world examples, were associated with more correct answers on the TOPKAM. Other studies have found positive correlations between CT skills tests and other measures of psychological misconceptions (McCutcheon et al. 1992; Kowalski and Taylor 2004). Bensley et al. (2014) also found that higher correct TOPKAM scores were positively correlated with scores on the Inventory of Thinking Dispositions in Psychology (ITDP) of Bensley (2021), a measure of the disposition to take a rational and scientific approach to psychology but were negatively correlated with an intuitive cognitive style.

Bensley et al. (2021) conducted a multidimensional study, assessing beginner psychology students starting a CT course on their endorsement of psychological misconceptions, recognition of thinking errors, CT dispositions, and metacognition, before and after CT instruction. Two classes received explicit instruction involving considerable practice in argument analysis and scientific reasoning skills, with one class receiving CT instruction focused more on recognizing psychological misconceptions and a second class focused more on recognizing various thinking errors. Bensley et al. assessed both classes before and after instruction on the TOPKAM and on the Test of Thinking Errors, a test of the ability to recognize in real-world examples 17 different types of thinking errors, such as confirmation bias, inappropriate use of the availability and representativeness heuristics, reasoning from ignorance/possibility, gambler’s fallacy, and hasty generalization (Bensley et al. 2021). Correct TOPKAM and TOTE scores were positively correlated, and after CT instruction both were positively correlated with the APS, the CT test of argument analysis skills.

Bensley et al. found that after explicit instruction of CT skills, students improved significantly on both the TOPKAM and TOTE, but those focusing on recognizing misconceptions improved the most. Also, those students who improved the most on the TOTE scored higher on the REI rational–analytic scale and on the ITDP, while those improving the most on the TOTE scored higher on the ITDP. The students receiving explicit CT skill instruction in recognizing misconceptions also significantly improved the accuracy of their metacognitive monitoring in estimating their TOPKAM scores after instruction.

Given that before instruction neither class differed in GPA nor on the SAT, a proxy for general cognitive ability, CT instruction provided a good accounting for the improvement in recognition of thinking errors and misconceptions without recourse to intelligence. However, SAT scores were positively correlated with both TOTE scores and APS scores, suggesting that cognitive ability contributed to CT skill performance. These results replicated the earlier findings of Bensley and Spero (2014) showing that explicit CT instruction improved performance on both CT skills tests and metacognitive monitoring accuracy while controlling for SAT, which was positively correlated with the CT skills test performance.

Taken together, these findings suggest that cognitive ability contributes to performance on CT tasks but that CT instruction goes beyond it to further improve performance. As the results of Bensley et al. (2021) show, and as discussed next, thinking errors and bias from heuristics are CT failures that should also be assessed because they are related to endorsement of unsubstantiated beliefs and cognitive style.

## 6. Dual-Processing Theory and Research on Unsubstantiated Beliefs

Consistent with DPT, numerous other studies have obtained significant positive correlations between intuitive cognitive style and paranormal belief, often using the REI intuitive–experiential scale and the Revised Paranormal Belief Scale (RPBS) of Tobacyk (2004) (e.g., Genovese 2005; Irwin and Young 2002; Lindeman and Aarnio 2006; Pennycook et al. 2015; Rogers et al. 2018; Saher and Lindeman 2005). Studies have also found positive correlations between superstitious belief and intuitive cognitive style (e.g., Lindeman and Aarnio 2006; Maqsood et al. 2018). REI intuitive–experiential thinking style was also positively correlated with belief in complementary and alternative medicine (Lindeman 2011), conspiracy theory belief (Alper et al. 2020), and with endorsement of psychological misconceptions (Bensley et al. 2014; Bensley et al. 2022).

Additional evidence for DPT has been found when REI R-A and NFC scores were negatively correlated with scores on measures of unsubstantiated beliefs, but studies correlating them with measures of paranormal belief and conspiracy theory belief have shown mixed results. Supporting a relationship, REI rational–analytic and NFC scores significantly and negatively predicted paranormal belief (Lobato et al. 2014; Pennycook et al. 2012). Other studies have also obtained a negative correlation between NFC and paranormal belief (Lindeman and Aarnio 2006; Rogers et al. 2018; Stahl and van Prooijen 2018), but both Genovese (2005) and Pennycook et al. (2015) found that NFC was not significantly correlated with paranormal belief. Swami et al. (2014) found that although REI R-A scores were negatively correlated with conspiracy theory belief, NFC scores were not.

Researchers often refer to people who are doubtful of paranormal and other unfounded claims as “skeptics” and so have tested whether measures related to skepticism are associated with less endorsement of unsubstantiated claims. They typically view skepticism as a stance towards unsubstantiated claims taken by rational people who reject them, (e.g., Lindeman and Aarnio 2006; Stahl and van Prooijen 2018), rather than as a disposition inclining a person to think critically about unsubstantiated beliefs (Bensley 2018).

Fasce and Pico (2019) conducted one of the few studies using a measure related to skeptical disposition, the Critical Thinking Disposition Scale (CTDS) of Sosu (2013), in relation to endorsement of unsubstantiated claims. They found that scores on the CTDS were negatively correlated with scores on the RPBS but not significantly correlated with either a measure of pseudoscience or of conspiracy theory belief. However, the CRT was negatively correlated with both RPBS and the pseudoscience measure. Because Fasce and Pico (2019) did not examine correlations with the Reflective Skepticism subscale of the CTDS, its contribution apart from full-scale CTDS was not found.

To more directly test skepticism as a disposition, we recently assessed college students on how well three new measures predicted endorsement of psychological misconceptions, paranormal claims, and conspiracy theories (Bensley et al. 2022). The dispositional measures included a measure of general skeptical attitude; a second measure, the Scientific Skepticism Scale (SSS), which focused more on waiting to accept claims until high-quality scientific evidence supported them; and a third measure, the Cynicism Scale (CS), which focused on doubting the sincerity of the motives of scientists and people in general. We found that although the general skepticism scale did not predict any of the unsubstantiated belief measures, SSS scores were a significant negative predictor of both paranormal belief and conspiracy theory belief. REI R-A scores were a less consistent negative predictor, while REI I-E scores were more consistent positive predictors, and surprisingly CS scores were the most consistent positive predictors of the unsubstantiated beliefs.

Researchers commonly assume that people who accept implausible, unsubstantiated claims are gullible or not sufficiently skeptical. For instance, van Prooijen (2019) has argued that conspiracy theory believers are more gullible (less skeptical) than non-believers and tend to accept unsubstantiated claims more than less gullible people. van Prooijen (2019) reviewed several studies supporting the claim that people who are more gullible tend to endorse conspiracy theories more. However, he did not report any studies in which a gullible disposition was directly measured.

Recently, we directly tested the gullibility hypothesis in relation to scientific skepticism (Bensley et al. 2023) using the Gullibility Scale of Teunisse et al. (2019) on which people skeptical of the paranormal had been shown to have lower scores. We found that Gullibility Scale and the Cynicism Scale scores were positively correlated, and both were significant positive predictors of unsubstantiated beliefs, in general, consistent with an intuitive–experiential cognitive style. In contrast, we found that scores on the Cognitive Reflection Test, the Scientific Skepticism Scale, and the REI rational–analytic scale were all positively intercorrelated and significant negative predictors of unsubstantiated beliefs, in general, consistent with a rational–analytic/reflective cognitive style. Scientific skepticism scores negatively predicted general endorsement of unsubstantiated claims beyond the REI R-A scale, but neither the CTDS nor the CTDS Reflective Skepticism subscale were significant. These results replicated findings from the Bensley et al. (2023) study and supported an elaborated dual-process model of unsubstantiated belief. The SSS was not only a substantial negative predictor, it was also negatively correlated with the Gullibility Scale, as expected.

These results suggest that both CT-related dispositions and CT skills are related to endorsement of unsubstantiated beliefs. However, a measure of general cognitive ability or intelligence must be examined along with measures of CT and unsubstantiated beliefs to determine if CT goes beyond intelligence to predict unsubstantiated beliefs. In one of the few studies that also included a measure of cognitive ability, Stahl and van Prooijen (2018) found that dispositional characteristics helped account for acceptance of conspiracies and paranormal belief beyond cognitive ability. Using the Importance of Rationality Scale (IRS), a rational–analytic scale designed to measure skepticism towards unsubstantiated beliefs, Stahl and van Prooijen (2018) found that the IRS was negatively correlated with paranormal belief and belief in conspiracy theories. In separate hierarchical regressions, cognitive ability was the strongest negative predictor of both paranormal belief and of conspiracy belief, but IRS scores in combination with cognitive ability negatively predicted endorsement of paranormal belief but did not significantly predict conspiracy theory belief. These results provided partial support that that a measure of rational–analytic cognitive style related to skeptical disposition added to the variance accounted for beyond cognitive ability in negatively predicting unsubstantiated belief.

In another study that included a measure of cognitive ability, Cavojova et al. (2019) examined how CT-related dispositions and the Scientific Reasoning Scale (SRS) were related to a measure of paranormal, pseudoscientific, and conspiracy theory beliefs. The SRS of Drummond and Fischhoff (2017) likely measures CT skill in that it measures the ability to evaluate scientific research and evidence. As expected, the unsubstantiated belief measure was negatively correlated with the SRS and a cognitive ability measure, similar to Raven’s Progressive Matrices. Unsubstantiated beliefs were positively correlated with dogmatism (the opposite of open-mindedness) but not with REI rational–analytic cognitive style. The SRS was a significant negative predictor of both unsubstantiated belief and susceptibility to bias beyond the contribution of cognitive ability, but neither dogmatism nor analytic thinking were significant predictors. Nevertheless, this study provides some support that a measure related to CT reasoning skill accounts for variance in unsubstantiated belief beyond cognitive ability.

The failure of this study to show a correlation between rational–analytic cognitive style and unsubstantiated beliefs, when some other studies have found significant correlations with it and related measures, has implications for the multidimensional assessment of unsubstantiated beliefs. One implication is that the REI rational–analytic scale may not be a strong predictor of unsubstantiated beliefs. In fact, we have recently found that the Scientific Skepticism Scale was a stronger negative predictor (Bensley et al. 2022; Bensley et al. 2023), which also suggests that other measures related to rational–analytic thinking styles should be examined. This could help triangulate the contribution of self-report cognitive style measures to endorsement of unsubstantiated claims, recognizing that the use of self-report measures has a checkered history in psychological research. A second implication is that once again, measures of critical thinking skill and cognitive ability were negative predictors of unsubstantiated belief and so they, too, should be included in future assessments of unsubstantiated beliefs.

## 7. Discussion

This review provided different lines of evidence supporting the claim that CT goes beyond cognitive ability in accounting for certain real-world outcomes. Participants who think critically reported fewer problems in everyday functioning, not expected to befall critical thinkers. People who endorsed unsubstantiated claims less showed better CT skills, more accurate domain-specific knowledge, less susceptibility to thinking errors and bias, and were more disposed to think critically. More specifically, they tended to be more scientifically skeptical and adopt a more rational–analytic cognitive style. In contrast, those who endorsed them more tended to be more cynical and adopt an intuitive–experiential cognitive style. These characteristics go beyond what standardized intelligence tests test. In some studies, the CT measures accounted for additional variance beyond the variance contributed by general cognitive ability.

That is not to say that measures of general cognitive ability are not useful. As noted by Gottfredson (2004), “g” is a highly successful predictor of academic and job performance. More is known about g and Gf than about many other psychological constructs. On average, g is closely related to Gf, which is highly correlated with working memory (*r* = 0.70) and can be as high as *r* = 0.77 (*r*^2^ = 0.60) based on a correlated two-factor model (Gignac 2014). Because modern working memory theory is, itself, a powerful theory (Chai et al. 2018), this lends construct validity to the fluid intelligence construct. Although cognitive scientists have clearly made progress in understanding the executive processes underlying intelligence, they have not yet identified the specific cognitive components of intelligence (Sternberg 2022). Moreover, theorists have acknowledged that intelligence must also include components beyond g, including domain-specific knowledge (Ackerman 2022; Conway and Kovacs 2018) which are not yet clearly understood,

This review also pointed to limitations in the research that should be addressed. So far, not only have few studies of unsubstantiated beliefs included measures of intelligence, but they have also often used proxies for intelligence test scores, such as SAT scores. Future studies, besides using more and better measures of intelligence, could benefit from inclusion of more specifically focused measures, such as measures of Gf and Gc. Also, more research should be carried out to develop additional high-quality measures of CT, including ones that assess specific reasoning skills and knowledge relevant to thinking about a subject, which could help resolve perennial questions about the domain-general versus domain-specific nature of intelligence and CT. Overall, the results of this review encourage taking a multidimensional approach to investigating the complex constructs of intelligence, CT, and unsubstantiated belief. Supporting these recommendations were results of studies in which the improvement accrued from explicit CT skill instruction could be more fully understood when CT skills, relevant knowledge, CT dispositions, metacognitive monitoring accuracy, and a proxy for intelligence were used.

## 8. Conclusions

Critical thinking, broadly conceived, offers ways to understand real-world outcomes of thinking beyond what general cognitive ability can provide and intelligence tests test. A multi-dimensional view of CT which includes specific reasoning and metacognitive skills, CT dispositions, and relevant knowledge can add to our understanding of why some people endorse unsubstantiated claims more than others do, going beyond what intelligence tests test. Although general cognitive ability and domain-general knowledge often contribute to performance on CT tasks, thinking critically about real-world questions also involves applying rules, criteria, and knowledge which are specific to the question under consideration, as well as the appropriate dispositions and cognitive styles for deploying these.

Despite the advantages of taking this multidimensional approach to CT in helping us to more fully understand everyday thinking and irrationality, it presents challenges for researchers and instructors. It implies the need to assess and instruct multidimensionally, including not only measures of reasoning skills but also addressing thinking errors and biases, dispositions, the knowledge relevant to a task, and the accuracy of metacognitive judgments. As noted by Dwyer (2023), adopting a more complex conceptualization of CT beyond just skills is needed, but it presents challenges for those seeking to improve students’ CT. Nevertheless, the research reviewed suggests that taking this multidimensional approach to CT can enhance our understanding of the endorsement of unsubstantiated claims beyond what standardized intelligence tests contribute. More research is needed to resolve remaining controversies and to develop evidence-based applications of the findings.

## Data Availability

This research did not involve collection of original data, and hence there are no new data to make available.
